# District Wellness Policy Nutrition Standards Are Associated with Healthier District Food Procurement Practices in the United States

**DOI:** 10.3390/nu12113417

**Published:** 2020-11-07

**Authors:** Elizabeth Piekarz-Porter, Julien Leider, Lindsey Turner, Jamie F. Chriqui

**Affiliations:** 1 Institute for Health Research and Policy, University of Illinois Chicago, Chicago, IL 60608, USA; jleide2@uic.edu (J.L.); jchriqui@uic.edu (J.F.C.); 2 Division of Health Policy and Administration, School of Public Health, University of Illinois Chicago, Chicago, IL 60612, USA; 3 College of Education, Boise State University, Boise, ID 83725, USA; lindseyturner1@boisestate.edu

**Keywords:** food procurement, child nutrition, district wellness policy, legal epidemiology, policy surveillance

## Abstract

Food procurement policies often exist to require that schools purchase foods with specific nutrient standards. Such policies are increasingly being used with the hope of improving access to healthier foods and beverages. Local wellness policies, required in any school district that participates in Federal Child Nutrition Programs, often contain specific nutrition standards that detail what can be sold to students during the school day. This study investigated the extent to which nutrition standards in wellness policies may be associated with healthier nutrition standards in district-level purchasing specifications. Cross-sectional data from the 2014–2015 school year for 490 school food authorities from 46 states and the District of Columbia were collected as part of the School Nutrition and Meal Cost Study and the National Wellness Policy Study. Survey-adjusted multivariable logistic regression models were computed to examine the association between district wellness policy nutrition standards and corresponding district food purchasing specifications. Results show that having a district wellness policy with corresponding nutrition standards and being in a rural area were associated with district food purchasing specifications for specific nutrients. These findings contribute to the literature to suggest that having a wellness policy with detailed nutrition standards may help to increase access to healthier foods and beverages.

## 1. Introduction

Under rules to participate in United States Child Nutrition Programs, local education agencies (LEAs; also referred to as school districts) are required to have local wellness policies that include nutrition standards for school meals and other foods sold that meet federal rules [1,2,3]. Such nutrition standards aim to provide healthier options to students during the school day, in an effort to combat consistently high rates of childhood overweight and obesity in the United States [4]. Percentages of children with unhealthy weights are higher in children of color and in rural America, adding to lifelong health disparities [4,5,6]. Prior research has shown that when wellness policies include strong, required provisions related to foods and snacks, students are likely to benefit by gaining access to healthier foods and beverages [7,8,9,10]. Yet, less is known about implementation of these policies by school food authorities (SFAs).

Even in school districts in which state law dictates strong nutrition standards and the SFA is striving to meet federal requirements, the SFA itself has to navigate how to find and purchase the appropriate products that meet the nutrition standards. At times, this task becomes overwhelming for local food service directors who are responsible for procuring improved products that are not always readily available [11,12].

A “food procurement policy” refers to a policy officially adopted by a state or local government or agency requiring that the food it purchases, provides, or makes available contains key nutrients at levels that are minimally aligned with standards established by public health authorities [13]. Food procurement policies specify details about the sources from which SFAs may purchase foods and beverages, and are integrally linked to the successful implementation of strong nutrition standards. Increasingly, food procurement policies address purchasing locally grown produce or aspire to healthier nutrition standards [14]. These policies stretch far beyond the school setting, and often exist for state and local government buildings, hospitals, colleges, day care centers, and assisted living facilities. 

Federal, state, and local governments in the United States routinely use indirect methods to impact behaviors related to public health. Efforts to combat obesity have utilized a variety of “carrot” (e.g., incentives such as healthy food certification) and “stick” (e.g., fiscal penalties for non-compliance with nutrition standards) approaches [15]. Nutrition standards involve direct regulation of SFAs, while healthy food procurement policies indirectly aim to shift the purchasing power of the government into a more positive direction.

Globally, there is growing evidence that food procurement policies can increase the availability and purchasing of healthier options [14,16]. When a set of school systems in Brazil was provided with guidelines that required purchasing from nearby family farms, the prevalence of healthy options at schools increased [17]. In Rome, procurement policies helped deliver higher quality foods and beverages to schools in which competitive bids were not based solely on cost but rather on what foods would be brought to the table [18]. In the United States, New York City was the first major municipality to adopt a nutrition policy for all foods purchased, served, or contracted for by City agencies [19]. In place since 2008, these standards apply to all city sites (e.g., schools, public hospitals, correctional facilities) and successful implementation was credited to several facilitating factors, including consistency across venues and the provision of technical assistance by registered dietitians [20]. Another study simulated results of reducing sodium intake in Los Angeles County through the use of a procurement policy and predicted reduced intake of sodium by consumers, fewer cases of uncontrolled hypertension, and reductions in direct health care costs [21].

However, food procurement policies themselves are not necessarily easy to implement. Leadership support, adequate vendor selections, and the assistance of dietitians in the implementation process have been found to be facilitators of healthy food procurement policies. However, when there aren’t enough choices or dietetic expertise, healthy food procurement becomes more challenging [22]. Moreover, research shows that rural communities and communities of color often lack access to healthier food choices and so may already be operating at a disadvantage [23,24,25,26,27,28]. 

Procurement policies provide the opportunity to create a healthier food environment and prompt the creation of new products that meet the improved standards. Although research has been done on food procurement policies and wellness policies independently, to our knowledge no study has looked at the creation and existence of both within the school space. Given the fact that wellness policies are required to include nutrition standards for school meals and other foods sold that meet federal rules and that food procurement policies are a primary mechanism for implementing the wellness policy provisions in practice, we sought to investigate our hypothesis that nutrition standards adopted as part of school district wellness policies will be associated with healthier nutrition standards included in district-level food purchasing practices. 

## 2. Materials and Methods

### 2.1. Data and Design

The School Nutrition and Meal Cost Study (SNMCS) was conducted in the 2014–2015 school year for the United States Department of Agriculture, Food and Nutrition Service [29]. Its purpose was to provide nationally representative data on school food service, and outcomes such as student dietary intakes, through collection of data from SFAs, schools, and students. This study utilized cross-sectional SFA-level data from SNMCS that are nationally representative of public SFAs that offer the National School Lunch Program, linked to data on district wellness policies from the National Wellness Policy Study (NWPS) [30,31]. NWPS data were linked by Mathematica Policy Research based on district identifiers, and de-identified data were returned to the University of Illinois Chicago for analysis. This study was deemed to “not involve human subjects” by the University of Illinois Chicago Institutional Review Board (protocol #2020-0448).

### 2.2. Measures

#### 2.2.1. SNMCS Outcome Measures

Data on food purchasing specifications were obtained from a question on the SNMCS SFA Director Survey asking, “Does your district use food purchasing specifications that include specific requirements for any of the following? Please do not include information requests to vendors or purchasing cooperatives as specific requirements in the specifications.” Eight specific nutritional characteristics were listed, as well as two other write-in options, with response options of “yes” or “no” for each. This analysis considered six potential requirements, for calories, sodium, total or added sugar, total fat, saturated fat, and trans fat. Requirements for whole grains were also considered for analyses, but due to the high prevalence of food purchasing specification requirements for whole grains (89.33%) and of corresponding district policies addressing whole grain-rich requirements (91.73%), we could not analyze this outcome.

#### 2.2.2. NWPS Measures

District policies limiting the calorie content of snacks or competitive entrées, regulating sodium content of snacks or competitive entrées, regulating sugar content, regulating fat content, limiting saturated fat, and limiting trans fat were coded separately for a la carte, vending machines, and school stores as part of the NWPS. Policies were coded separately by school level (elementary, middle, and high school). For purposes of the analyses herein linking to the SNMCS food purchasing specification requirement outcomes, separate policy measures were computed classifying each district as having no policy, a weak or suggested policy, or a required policy on each of these six items, based on the strongest policy (e.g., required or weak policy) present across the three venues and all school levels. Required policies included language with definitive limits on the nutrients evaluated here, and/or rose to the level of the Smart Snacks nutrient standards.

#### 2.2.3. Contextual Characteristics

District and SFA characteristics were obtained from the National Center for Education Statistics, the 2011 Census Bureau Small Area Income and Poverty Estimates (SAIPE) school district file, and the SFA Verification Summary Report 2012–2013 [32,33,34,35]. The district racial/ethnic distribution was captured through continuous variables for the percentages of students who were non-Hispanic white, non-Hispanic black, and Hispanic; the district child poverty rate was categorized as ≥20% versus <20%; district locale was categorized as large to mid-size city, suburban, rural, or township; and SFA size was categorized as <1000, 1000–5000, or >5000 students. Census region was categorized based on Census definitions as South, West, Midwest, and Northeast [36]. To allow adequate power in analyses, groupings for several categorical district characteristics were combined as follows: large to mid-size city, suburban, or rural/township (locale); >5000 versus ≤5000 students (size); and South versus non-South (region).

### 2.3. Study Sample

The SFA Director Survey was completed by 518/548 SFAs (95.7% weighted response rate). Out of these 518 SFAs, three SFAs were missing data on one or more district characteristics needed for analyses, 22 additional SFAs were missing NWPS district policy data, and of the remaining SFAs, three were missing data on all six food purchasing specification outcomes, leaving 490 SFAs in 46 states and the District of Columbia (DC) in the analytical sample. Sample sizes for individual analyses ranged from 486–489 SFAs due to missing data for specific outcomes. Due to differences in the analytical sample, descriptive statistics in this paper may differ from those in the SNMCS report [37]. 

### 2.4. Data Analysis

Six separate multivariable logistic regressions were computed at the SFA level. A separate, multivariable logistic regression model was fitted for each of the six district food purchasing specification requirements listed in Table 1 (i.e., each requirement was a separate outcome in each of the six models). The key independent variable for each model was the corresponding district wellness policy nutrition standard noted in Table 2. For example, the first regression model examined the district food purchasing specification requirement variable of “calories”; this binary variable (1 = had requirement, 0 = no requirement) was regressed on district wellness policy provisions for “calorie content of snacks/entrees” (categorical variable: 0 = no policy (ref), 1 = weak/suggested/encouraged policy, 2 = required policy). Each regression model controlled for all other district characteristics noted in Table 1 as follows: district race/ethnicity (three separate continuous variables; one each for percent non-Hispanic white, percent non-Hispanic black, and percent Hispanic); district locale (categorical variable: 1 = urban (ref), 2 = suburban, 3 = rural/township); district-level child poverty rate (binary variable: 0 = <20% (ref), 1 = 20% or greater); SFA size (binary variable: 0 = >5000 students (ref), 1 = ≤ 5000 students); and Census region (binary variable: 0 = South (ref), 1 = Non-South). Adjusted prevalence estimates were computed from these models. Analyses accounted for the survey design and weights, and were conducted in Stata/SE (version 15.1, StataCorp LP, College Station, TX, USA; 2016).

## 3. Results

### 3.1. SFA and District Characteristics

Table 1 shows the survey-weighted sample characteristics. District food purchasing specifications were relatively prevalent, ranging from 61% of SFAs restricting total or added sugars to 84% of SFAs restricting trans fats. The districts were predominantly white (approximately 70% of district students on average), located in rural areas or small townships (66%), and had a child poverty rate of less than 20% (60%). Most (85%) of the SFAs were small (≤5000 students) and located in non-Southern regions of the country (74%). 

### 3.2. Prevalence of District Nutrient Standards

The majority of district wellness policies required or suggested standards for the nutrients of interest (Table 2). An additional one-quarter to one-third (or thereabouts) did not address specific nutrient standards in their district policies (Table 2). 

### 3.3. Factors Associated with District Food Purchasing Specification Requirements

Results of the multivariable logistic regressions examining the factors associated with district food purchasing specification requirements are presented in Table 3. Overall, the two factors consistently associated with district food purchasing specifications were having a district policy and being in a rural area. Specifically, district food purchasing specifications regarding saturated fats (AOR: 3.83, 95% CI: 1.61, 9.10) and total or added sugars (AOR: 2.77, CI: 1.20, 6.39) were more common in districts with policies that encouraged such provisions as compared to no policy at all. Interestingly, districts located in rural areas or townships were more likely to have food purchasing specification standards for calories (AOR: 2.89, CI: 1.21, 6.90) and trans fat (AOR: 3.06, CI: 1.07, 8.81) as compared to districts located in large-to-mid-size cities.

## 4. Discussion

Many children eat two to three meals per day at school. This is particularly true in lower income communities in which students may receive breakfast, lunch, an after-school snack, and/or dinner. Improving the nutritional quality of foods and beverages served becomes key in supporting the healthy development of children. Prior research shows that strong district wellness policy provisions are often associated with implementation of healthy nutrition standards [7,8,9,10]. Similarly, food procurement policies have the potential to increase access to healthy foods and beverages at school [14,16]. This study finds that districts were more likely to have procurement policies on saturated fats and sugars when the wellness policy at least suggested or encouraged limits on the same nutrients. At the same time, rural school districts were more likely to include purchasing specifications for calories and trans fat when compared to urban districts. 

The fact that most district wellness policies required specific nutrient standards is not surprising given that the study was conducted during the first year that the U.S. Department of Agriculture’s Smart Snacks in Schools standards took effect and specified nutrient standards for all foods sold at school outside of the meal programs [1]. Furthermore, the NWPS coding scheme gave credit for having a required policy in each nutrient standard if the district policy generally required adherence to the federal Smart Snacks standards [31]. As a result, it is possible that some districts in which there is a general reference to the federal standards could further revise their policies to include the limits on specific nutrients and add clarity in the next round of revisions. 

Districts were more likely to have procurement policies on saturated fats and sugars when mentioned in the wellness policy, but wellness policies on other nutrients were not significantly associated with procurement policies. Solid fats and added sugars (SoFAS) have been termed by the 2010 Dietary Guidelines for Americans as “empty calories” [38]. Consistently, research has identified that schools often provide easy access to SoFAS [39,40], but that students who attend schools in which SoFAS are limited were less likely to consume empty calories [7]. Because SoFAS have been identified as key in attaining the goal of healthy weights in children, it is not surprising that associations were found for saturated fats and sugars but not for other nutrients.

All LEAs that participate in the federal Child Nutrition Programs must adhere to the Smart Snacks standards [1,2]. However, district wellness policies tend to be updated on a triennial basis [30], similar to the cycle for administrative reviews of food service programs [41]. Consequently, although the federal standards went into effect in the 2014–15 school year (the same year that the data collection for this study took place), some district policies may not yet have been revised to reflect the Smart Snacks standards. Thus, the finding that policies that encouraged or suggested specific nutrient standards were associated with food purchasing practices is encouraging because these “weaker” policies likely will have only strengthened over time as the policies “caught up” to the federal requirements. Even so, other studies have likewise found associations with policies that encourage practices [9,42] and this study’s finding reiterates that any policy can be a step in the right direction.

Still, to date, many policies that fail to take into account socioeconomic inequities have been found to have neutral impacts on closing the gap between inequities in obesity [43]. In 2012, rural children were significantly more likely to be obese or overweight than were their urban counterparts [6]. During the year of this study, 18.6% of students in the United States attended school in a rural SFA [44]. At the same time, 23.7% of rural children were estimated to be living in poverty ($24,008 for a family of four) [45]. Such statistics further the need for increased district policy in this space, especially with the potential to increase the likelihood of procurement standards and an improvement in access to healthy foods and beverages.

The regression models showed that purchasing standards limiting calories and trans fat were more common in rural districts than in urban districts. While it unclear as to why this pattern occurred, possibilities relate to the ways in which district procurement standards are written (i.e., possible variations in technical assistance within states, the use of model purchasing standards by states or regions with more rural schools), or potential alignment with purchasing based on local suppliers’ product availability. Although it is not possible to confirm the reasons for these variations by rurality, it is clear that rural school districts face unique challenges to the delivery and establishment of Child Nutrition Programs that conform to USDA requirements. Rural school districts provide service to fewer students, tend to experience a higher cost of food (often based on transport and logistics), work with smaller food service staff, and often include a highly dispersed network of schools [46,47]. This study finds that rural school districts were more likely to have food procurement policies regulating calories or trans fats than urban school districts. Given the challenges that rural school districts face, particularly in light of issues physically procuring food, it is likely that such policies pave the way for a better and/or more streamlined relationship between food service directors and food suppliers. As a result, rural school districts may find even greater benefits to adopting nutrition standards in district wellness policies and local procurement standards to assist in food purchasing.

With the growing evidence that procurement policies provide greater access to healthier foods and beverages [14,16], continued adoption of nutrition-related wellness policy provisions could help to narrow the inequities in food access among children in the United States, particularly those in rural America. Previous studies have found mixed results when it comes to whether procurement policies lead to lower body mass indices [14], but future studies could evaluate the impact of food procurement and availability on other health outcomes.

### Limitations and Areas for Future Research

The findings presented herein should be considered with the following limitations in mind. First, this was a cross-sectional analysis of district wellness and food procurement policies conducted during one school year. USDA’s Smart Snacks standards first took effect during that school year, so it is likely that there has been continued strengthening of both district wellness and procurement policies and practices since the time of data collection. Future studies should explore how the nutrition standards and procurement policy landscape has changed in the ensuing years since Smart Snacks took effect. Second, this was a correlational study and, therefore, causation cannot be inferred. Third, although we examined the relationship between district policy provisions and district-reported food procurement nutrient standards in practice, SNMCS data did not allow us to examine the final purchasing decisions of school districts (or schools) and what foods were available. Further research is needed to understand what drives district procurement decisions, and the role of district, state, and federal policies in those decisions. Finally, future research should include both quantitative and qualitative methods to understand the breadth of factors influencing food procurement and how those factors vary across a wide range of districts, particularly rural versus non-rural districts given the findings from this study. 

## 5. Conclusions

There is a constant struggle to ensure that children have access to healthy foods and beverages during the school day. District wellness policy nutrition standards and detailed procurement policies work in tandem to contribute to the same overall goal of improving the eating habits of children by making the choices available to them at school better. This study finds that for some nutrient standards, having a wellness policy that even suggests or encourages specific nutrients is associated with the district having a corresponding food procurement policy. Similarly, rural school districts are more likely to adopt procurement policies that regulate what food can be purchased. Both findings point to the continued importance of regulating food sold at school through local policymaking. 

## Figures and Tables

**Table 1:** Survey-weighted sample characteristics.

Variable	% or Mean
District Food Purchasing Specification Requirements	
Calories	69.71
Total fat	72.59
Saturated fat	73.41
Trans fat	84.30
Sodium	78.51
Total or added sugar	61.15
District Race/Ethnicity	
Percent NH White (Mean)	69.30
Percent NH Black (Mean)	10.96
Percent Hispanic (Mean)	13.35
District Locale	
Urban	12.30
Suburban	21.44
Rural/Township	66.26
District Child Poverty Rate	
<20%	60.37
20% or greater	39.63
SFA Size	
≤5000 students	85.24
>5000 students	14.76
Region	
South	25.83
Non-South	74.17

NH, non-Hispanic; SFA, school food authority. *n* = 486–490 SFAs, due to item-specific missing data. Data on district food purchasing specification requirements were obtained from the School Nutrition and Meal Cost Study. District and SFA characteristics were obtained from the National Center for Education Statistics, the 2011 Census Bureau Small Area Income and Poverty Estimates school district file, and the SFA Verification Summary Report 2012–2013.

**Table 2:** District wellness policy nutrient standards prevalence.

District Wellness Policy Nutrient Standard	District Wellness Policy Nutrient Standard Strength		
	No Policy	Weak/Suggested/ Encouraged Policy	Required Policy
Limits calorie content of snacks/entrées	36.87	8.98	54.15
Regulates fat content	24.05	16.62	59.33
Limits saturated fat	25.45	16.75	57.80
Limits trans fat	33.42	13.61	52.97
Regulates sodium content of snacks/entrées	32.25	15.06	52.68
Regulates sugar content	26.55	15.68	57.77

SFA, school food authority. *n* = 490 SFAs included in the analytical sample. Data on district wellness policies were obtained from the National Wellness Policy Study.

**Table 3:** Logistic regression results for the association between selected district wellness policy nutrient standards and district food purchasing specification requirements.

Variable	Calories	Total Fat	Saturated Fat	Trans Fat	Sodium	Total or Added Sugar
	AOR (95% CI)	AOR (95% CI)	AOR (95% CI)	AOR (95% CI)	AOR (95% CI)	AOR (95% CI)
District Wellness Policy Categorization						
No Policy (Ref)	1.00 (1.00, 1.00)	1.00 (1.00, 1.00)	1.00 (1.00, 1.00)	1.00 (1.00, 1.00)	1.00 (1.00, 1.00)	1.00 (1.00, 1.00)
Weak/Suggested Policy	1.02 (0.42, 2.45)	2.49 (0.93, 6.64)	3.83 ** (1.61, 9.10)	2.19 (0.92, 5.22)	1.48 (0.64, 3.44)	2.77 * (1.20, 6.39)
Required Policy	0.93 (0.55, 1.57)	1.64 (0.89, 3.03)	1.83 (0.99, 3.39)	1.46 (0.77, 2.76)	1.28 (0.72, 2.29)	1.82 (0.90, 3.68)
District Race/Ethnicity						
Percent NH White	0.98 (0.96, 1.00)	0.98 (0.96, 1.01)	0.99 (0.97, 1.01)	0.99 (0.97, 1.02)	0.99 (0.97, 1.01)	0.98 (0.96, 1.01)
Percent NH Black	1.00 (0.98, 1.03)	1.00 (0.97, 1.02)	1.01 (0.98, 1.03)	1.02 (0.99, 1.04)	1.00 (0.98, 1.03)	1.00 (0.98, 1.03)
Percent Hispanic	1.00 (0.98, 1.02)	0.99 (0.97, 1.02)	1.00 (0.98, 1.03)	1.00 (0.98, 1.03)	0.99 (0.97, 1.02)	0.99 (0.97, 1.02)
District Locale						
Urban (Ref)	1.00 (1.00, 1.00)	1.00 (1.00, 1.00)	1.00 (1.00, 1.00)	1.00 (1.00, 1.00)	1.00 (1.00, 1.00)	1.00 (1.00, 1.00)
Suburban	2.14 (0.93, 4.91)	1.31 (0.57, 3.00)	1.16 (0.49, 2.77)	1.94 (0.75, 5.00)	1.21 (0.48, 3.01)	1.83 (0.78, 4.28)
Rural/Township	2.89 * (1.21, 6.90)	1.86 (0.77, 4.50)	2.36 (0.93, 5.95)	3.06 * (1.07, 8.81)	1.55 (0.60, 4.02)	1.83 (0.76, 4.42)
District Child Poverty Rate						
<20% (Ref)	1.00 (1.00, 1.00)	1.00 (1.00, 1.00)	1.00 (1.00, 1.00)	1.00 (1.00, 1.00)	1.00 (1.00, 1.00)	1.00 (1.00, 1.00)
20% or greater	1.17 (0.67, 2.07)	1.21 (0.69, 2.11)	1.47 (0.81, 2.66)	1.32 (0.68, 2.56)	1.05 (0.58, 1.90)	1.30 (0.62, 2.71)
SFA Size						
>5000 Students (Ref)	1.00 (1.00, 1.00)	1.00 (1.00, 1.00)	1.00 (1.00, 1.00)	1.00 (1.00, 1.00)	1.00 (1.00, 1.00)	1.00 (1.00, 1.00)
≤5000 students	1.23 (0.68, 2.24)	1.10 (0.60, 2.01)	0.88 (0.48, 1.64)	0.93 (0.44, 1.99)	1.22 (0.64, 2.33)	1.30 (0.71, 2.40)
Region						
South (Ref)	1.00 (1.00, 1.00)	1.00 (1.00, 1.00)	1.00 (1.00, 1.00)	1.00 (1.00, 1.00)	1.00 (1.00, 1.00)	1.00 (1.00, 1.00)
Non-South	0.91 (0.43, 1.90)	0.89 (0.45, 1.74)	1.22 (0.59, 2.54)	0.86 (0.38, 1.90)	0.52 (0.24, 1.13)	1.39 (0.64, 3.03)
N of schools in analysis	489	489	486	489	489	487
Adjusted Prevalence of District Food Purchasing Specification Requirement by District-level Policy Categorization						
No Policy	70.48%	63.66%	62.29%	80.29%	75.47%	49.66%
Suggested Policy	70.89%	81.03%	85.76%	89.76%	81.88%	72.30%
Required Policy	68.99%	73.96%	74.65%	85.46%	79.65%	63.55%

AOR, adjusted odds ratio; CI, confidence interval; NH, non-Hispanic; Ref, referent category; SFA, school food authority. Data on district food purchasing specification requirements were obtained from the School Nutrition and Meal Cost Study. Data on district wellness policies were obtained from the National Wellness Policy Study. District and SFA characteristics were obtained from the National Center for Education Statistics, the 2011 Census Bureau Small Area Income and Poverty Estimates school district file, and the SFA Verification Summary Report 2012–2013. Separate multivariable logistic regressions were run for each district food purchasing specification requirement outcome. For each model, we examined the relationship between the corresponding district wellness policy nutrient standard and the district food purchasing specification requirement outcome. For example, in the “calories” model, we examined whether the odds of the SFA procurement practices addressing calories varied by whether the district wellness policy required, encouraged, or did not address calories for entrées/snacks along with other district and SFA-level characteristics. * *p* < 0.05 ** *p* < 0.01.
